# Predicting the Strength of Cohesive and Adhesive Interparticle Interactions for Dry Powder Inhalation Blends of Terbutaline Sulfate with α-Lactose Monohydrate

**DOI:** 10.1021/acs.molpharmaceut.3c00292

**Published:** 2023-09-08

**Authors:** Cai Y. Ma, Thai T. H. Nguyen, Parmesh Gajjar, Ioanna D. Styliari, Robert B. Hammond, Philip J. Withers, Darragh Murnane, Kevin J. Roberts

**Affiliations:** † Centre for the Digital Design of Drug Products, School of Chemical and Process Engineering, University of Leeds, Leeds, LS2 9JT, U.K.; ‡ School of Materials, Henry Royce Institute, University of Manchester, Oxford Road, Manchester, M13 9PL, U.K.; § School of Life and Medical Sciences, University of Hertfordshire, College Lane, Hatfield, AL10 9AB, U.K.

**Keywords:** interparticle interactions, molecular modeling, terbutaline sulfate, α-lactose monohydrate, powder inhalation formulations, X-ray computed tomography

## Abstract

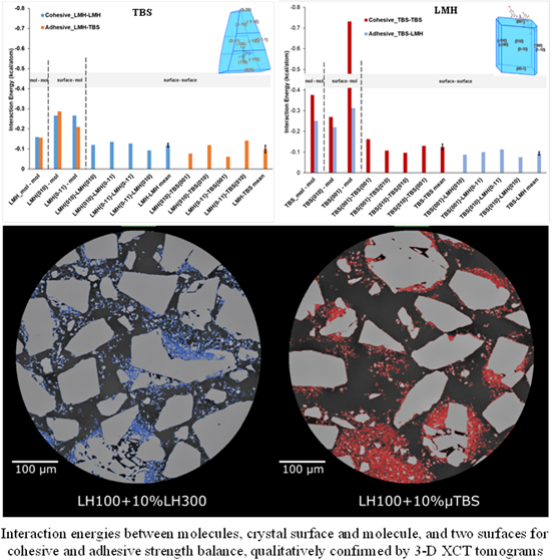

Grid-based systematic search methods are used to investigate molecule–molecule, molecule–surface, and surface–surface contributions to interparticle interactions in order to identify the crystal faces that most strongly affect particle behavior during powder blend formulation and delivery processes. The model system comprises terbutaline sulfate (TBS) as an active pharmaceutical ingredient (API) and α-form lactose monohydrate (LMH). A combination of systematic molecular modeling and X-ray computed tomography (XCT) is used to determine not only the adhesive and cohesive interparticle energies but, also the agglomeration behavior during manufacturing and de-agglomeration behavior during delivery after inhalation. This is achieved through a detailed examination of the balance between the adhesive and cohesive energies with the XCT results confirming the blend segregation tendencies, through the particle–particle de-agglomeration process. The results reveal that the cohesive interaction energies of TBS–TBS are higher than the adhesive energies between TBS and LMH, but that the cohesive energies of LMH–LMH are the smallest between molecule and molecule, molecule and surface, and surface and surface. This shows how systematic grid-search molecular modeling along with XCT can guide the digital formulation design of inhalation powders in order to achieve optimum aerosolization and efficacy for inhaled medicines. This will lead to faster pharmaceutical design with less variability, higher quality, and enhanced performance.

## 1 Introduction

Despite significant advances in drug discovery that have resulted in part from enhanced molecular modeling, the translation of discovery compounds into marketed products remains a lengthy process which is often referred to as low development productivity.^1,2^ One of the primary reasons behind this low productivity is the high, but necessary, quality requirements ensuring that a drug product is efficacious, but not harmful to patients. Improving pharmaceutical productivity rates will not only boost efficiency but also result in better care options. The challenge to this is ensuring that quality is not compromised.

Quality by design (QbD) has become important for the pharmaceutical industry, adopted by regulatory agencies such as the USA Food and Drug Administration and the European Medicines Agency to improve product quality by integrating quality considerations throughout the continuous design and development of a product.^3^ The main tenets of pharmaceutical QbD are:

(1) Defining and achieving product quality specifications that are based on clinical performance;
(2) Using product and process design to increase process capability and reduce product variability and defects.

Most pharmaceutical ingredients are polycrystalline solids which are formulated into dosage forms such as tablets, aerosols, capsules, suspensions, or suppositories.^4^ Thus, achieving a fundamental understanding of the key physicochemical and crystalline properties of such solids alongside their impact on processing, performance, and structure is vital.^5^ In particular, surface physicochemical interactions play an important role in processing, with blending, filling, and tableting all affected by the way crystals and molecules interact with each other.^6^ Equally, surface interactions can play a pivotal role in performance, for example, in the case of dry powder inhalers where the colloidal interactions between micron-sized drug particles promote cohesive agglomeration, and dominate adhesion to excipients and packaging, consequently defining the aerosolization performance.^7^ As well as their utility in drug design and discovery, molecular-based computational design tools can be integrated into R&D workflows in order to design-in and ensure product quality in pharmaceutical products, forming an integral part of Industry 4.0 approaches. The further development of the “*digital twin*,” i.e., a realistic but in silico replica of a material or process with quantitative validation, can form a bridge between the physical and virtual worlds and has accelerated progress in this area. Within the pharmaceutical sector, for example, information gained from an analysis of the crystal structures of ingredients together with in situ monitoring of their processing behavior can be used to adapt the predictive and process control models used in product manufacture. Further development of digital twinning for deployment in formulation design in order to improve clinical performance and hence product safety, efficacy, and cost-effectiveness offers distinct advantages. The application of digital approaches within the pharmaceutical sector demands the development and validation of predictive models. In this respect, characterizing the crystal morphology and hence the surface chemistry of the surfaces of the ingredients is important, as changes in the relative surface areas of different crystal habit surfaces can affect the overall chemical and physical properties expressed during processing. A cascade of process and product-based model can be built up from the molecular and crystallographic levels, working upward in scale through a multiscale approach aimed to simulate process behavior and product performance in order to control and ensure product quality.

At the molecular level, understanding the spatial arrangements of molecules together with the intermolecular forces holding the formulated ingredient particles together makes it possible to predict, control, and design not only the physicochemical properties of the crystalline ingredients but also the surface-terminated intermolecular interactions (extrinsic synthons) (see e.g., refs (200), (8)) that determine the nature of interparticle interactions important in formulations, notably the cohesive interactions within ingredient powders and the adhesive interactions between the different ingredients within a formulated product. Such synthonic engineering techniques have been used to predict the crystal morphology and surface chemistry, the mediation of crystal growth by additives or impurities, hydration, the stability of mixtures of crystals, and the physical and chemical properties of the formulated compounds (see, for example, refs (8−18)). This approach can be used, for example, in the design of crystallization processes and also to facilitate the subsequent formulation. Systematic grid-based searching^19^ has also been applied to investigate solvent wetting of crystal surfaces in which a single molecule is used as the probe. Similarly, in a related approach, methods have been developed to investigate molecule–molecule interaction energies between two crystal surfaces to produce energy maps.^10,19−21^ Ramachandran et al.^18^ investigated the functional relevance of synthonic modeling to the formulation of inhalation powders by assessing cohesive/adhesive forces between molecules for three APIs (fluticasone propionate, budesonide, and salbutamol base) and one excipient, α-lactose monohydrate, to respective simulated crystal surfaces. Nguyen et al.^17^ developed a digital workflow for predicting the physicochemical properties of relevance to the formulation of terbutaline sulfate by relating the intermolecular (synthonic) features to its crystal morphology and surface chemistry. Through this, the calculation of all of the individual crystal surfaces led to the calculation of surface area weighted estimation of the whole crystal particle surface energy and its dispersive and polar subcomponents providing, essentially, a virtual surface energy analysis system. The calculated surface energies of terbutaline sulfate (TBS) crystals using molecule–surface systematic search approaches^17^ were found to be in good agreement with experimental measurements using inverse gas chromatography (IGC), validating the predictive strength of the modeling. For example, the predicted surface energy of {001} crystal surfaces of 109 mJ/m^2^ is very close to the experimentally measured value of 103.3 mJ/m^2^ at low IGC surface coverage of micronized TBS (μTBS), revealing it to be capable of probing the highest surface energy “hot spots” for interparticle bonding. The molecular modeling also identified the low-surface-energy sites with agreement to IGC at higher surface coverage,^17^ including at surface coverages beyond that achievable with the experimental technique.

For α-form lactose monohydrate (LMH), the predicted particle surface energies were calculated based on the attachment energy model and crystal surface areas as predicted from the morphological simulation together with the experimentally determined surface energies of LMH being measured by IGC for comparisons.^22^ The calculated particle surface energy for LMH^22^ was found to be comparable to the previously published surface energy (77.6 mJ/m^2^)^18^ for coarse-grade of LMH and also the experimentally measured one (with IGC surface coverage from 1 to 20%) for sieved lactose (Lactohale 100).^22^ More extensive data for the latter will be available in a paper^22^ under preparation. Therefore, for both μTBS and LMH, the modeled surface energetics predictions have been validated quantitatively, as well as qualitatively for the full heterogeneity of surface energy distributions including both “active sites” and the lower-energy surface areas, which constitute the bulk of the particle surface.

Since the important behavior in many pharmaceutical products involves interactions between crystal facets of particles, the latter approach was developed further to predict and quantify the crystal facet interactions at the particle–particle level within a powder bed of hexamine crystals.^23,24^ A key advance would be the ability to model and characterize the interactions between the different crystal facets exposed on the surfaces of the different chemical formulation entities. Here, we link the modeling of interparticle interactions within a binary formulation mixture to the product performance. Crystal–crystal interactions of the classical dry powder inhaler blend comprising the API terbutaline sulfate and excipient α-form lactose monohydrate, used in inhalation devices such as the Bricanyl Turbohaler,^25^ are derived through synthonic modeling. As such, predictions are made for the adhesive and cohesive energy balances within this model powder blend as that affects agglomeration behavior during manufacturing and de-agglomeration during drug delivery. These predictions for the formation of a cohesively balanced (i.e., segregated) or adhesively balanced blend are assessed and confirmed alongside a qualitative comparison of representative inhalation blends manufactured by high-shear blending analyzed in 3D, using emerging methods in micro-X-ray computed tomography.^26^

## 2 Material and Methods

### 2.1 Materials

The model inhalation blend selected for this study was a combination of α-form lactose monohydrate as the excipient and terbutaline sulfate as the API. Synthonic modeling of intermolecular and interparticulate interaction forces was performed for these two compounds.

**Figure 1:**
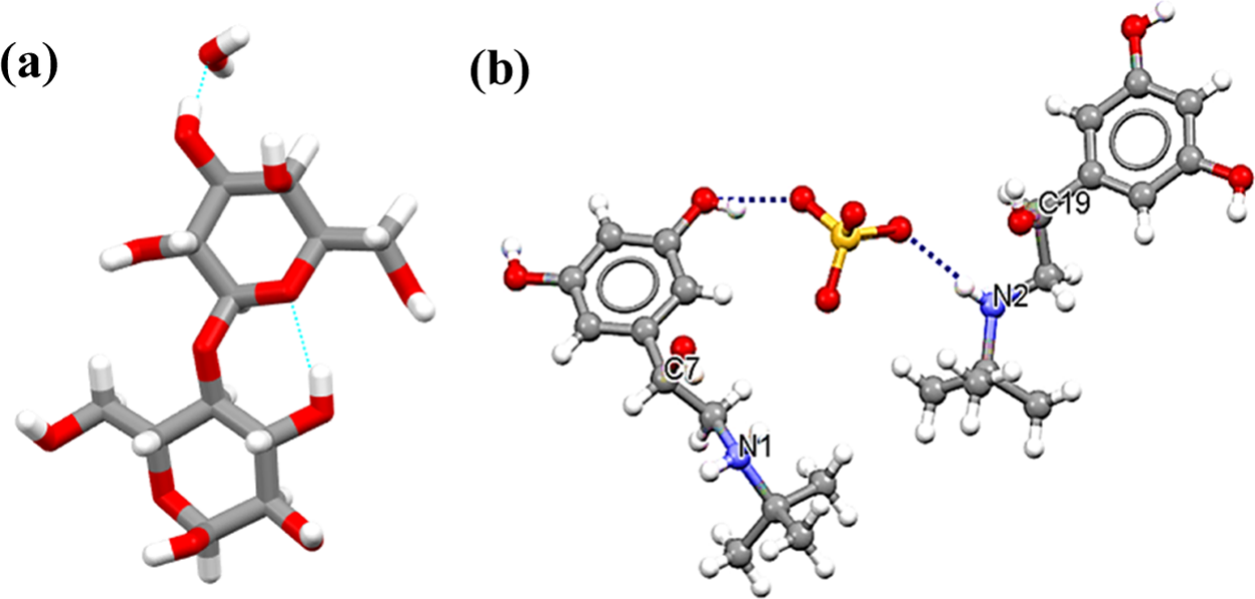
Molecular structures of (a) α-form lactose monohydrate (excipient) and (b) terbutaline sulfate (API) 2[C_12_H_20_NO_3_]^+^·S_4_^2–^ with dotted lines indicating hydrogen-bonding interactions.

The crystal structures of these materials (ZIVKAQ,^27^ LACTOS11^28^) were taken from the Cambridge Structural Database. The molecular structures of TBS and α-form LMH are shown in Figure 1. The stable anhydrate form B of TBS has a triclinic crystal structure with space group *P*1̅ and unit cell parameters: *a* = 9.968 Å, *b* = 11.207 Å, *c* = 13.394 Å, α = 100.86°, β = 104.42°, γ = 101.63°, with a tri-ionic (2 cation and 1 anion) asymmetric unit.^27^ The structure of the monohydrate form of α-Lactose is monoclinic with space group *P*2_1_ and unit cell parameters: *a* = 4.783 Å, *b* = 21.54 Å, *c* = 7.7599 Å, β = 105.911°.^28^

### 2.2 Computational Modeling

The interparticle compatibility of the blended materials was addressed with systematic atom–atom grid-search methods with empirical force fields using the SystSearch software in 3 modes: molecule–molecule,^10,20,21^ molecule–surface,^17−19^ and surface–surface^23^ modes.

#### 2.2.1 Intermolecular Compatibility

**Figure 2:**
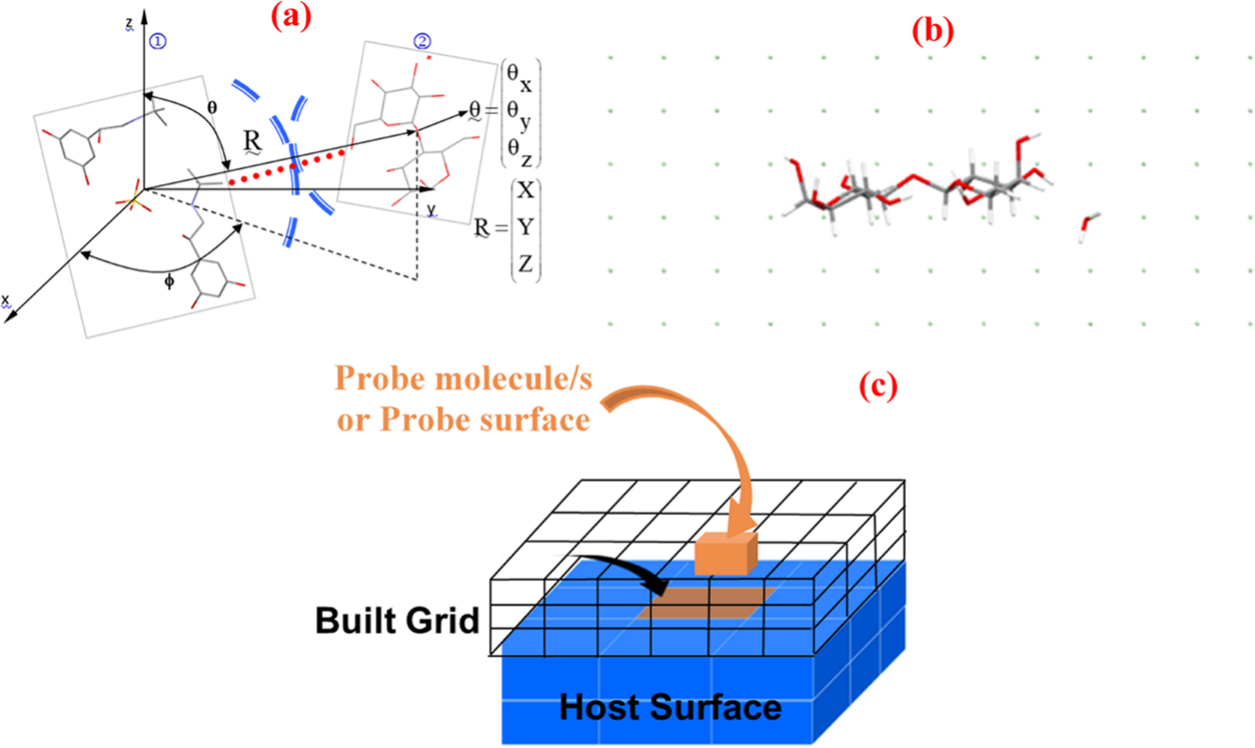
Schematics illustrating (a) the molecule (TBS)–molecule (LMH) grid-based systematic search; (b) orthogonal grid search built around the host molecule α-form LMH; and (c) surface–molecule and surface–surface grid-based search.

The most favored binding sites between two molecules^11,20,21,29^ were identified using the systematic search with stationary and mobile phases being single molecules, dimers, or surfaces and collectively referred to as a “body”. In this approach, the stationary body (host) is fixed, while the mobile body moves around the host either on a user-defined 3D grid or within orthogonal grids for intermolecular systematic search. At each grid point, the mobile phase can also rotate about three angles. At each point and rotation, the nonbonded intermolecular interaction energies between the host and the probe are calculated using the Dreiding potential parameters.^30^ By way of an example, the principle of the molecule–molecule systematic search^21,31^ and the orthogonal 3D grid built around the host molecule α-form LMH are shown in Figure 2a,b, respectively. The search results were ranked based on the intermolecular energy, with the top ranked dimers examined further.

#### 2.2.2 Molecule–Surface Compatibility

The grid-based search method was applied to a molecule-cleaved crystal surface system, i.e., the host molecule (1) in Figure 2a) was replaced by a crystal slab and a 3D grid built on the top of a cleaved crystal surface (Figure 2c). Similarly, the interactions between the probe molecule and the cleaved crystal face were calculated at each point within the 3D grid domain and ranked based on the interactional energy. Then, the interactional energy was used to calculate the adhesive strengths of TBS with LMH and the cohesive strengths between TBS–TBS and LMH–LMH.

#### 2.2.3 Particle Surface–Surface Compatibility

The surface–surface systematic grid search is a further extension of the molecule–crystal surface search described above. As shown in Figure 2c, in the surface–surface search, the probe is a cleaved crystal surface containing a number of molecules (probe surface). At each grid point, the interactional energy between the two surfaces is calculated with the probe surface being rotated in three dimensions. The ranked energy is used to determine the adhesive and cohesive strengths between these two surfaces, i.e., TBS–TBS and LMH–LMH for cohesion and TBS with LMH for adhesion. In these simulations, both probe molecules and surface slabs are treated as “rigid” bodies. The molecular structures for probe molecules are shown in Figure 1, and the probe surface slabs were created from TBS or LMH molecules in the first molecular layer of the corresponding crystal surface. As the size of the probe is very different for the three types of searches, the calculated interaction (binding) energies were represented by normalization with respect to the number of atoms contained in the probe.

#### 2.2.4 Crystal Surface Slab Construction and Probe Surface Selection

The crystal surface slab selected was a representation of the termination of the bulk crystal structure by the habit plane in the crystal morphology as defined by its Miller index (*h k l*). In this, the rational way is to align the normal direction to the reciprocal lattice plane (*h k l*) of the crystal structure with one of the Cartesian axis directions^19^ of the simulation frame. A set of conditions can be computed to meet the condition that the angle between the transformed, direct-space unit cell vectors in the transformed direct lattice is equal to 90° (for further details, see Rosbottom et al.^19^). To select the most stable surface termination, the attachment energy for the selected surface was calculated using HABIT98^32,33^ using a method outlined in previous publications.^34,35^ The most stable surface termination was determined by shifting the surface termination by 0.1 d-spacing (*d*_*hkl*_) through one full d-spacing, and the termination with the lowest absolute value of attachment energy was assumed to be the most stable surface termination.^19^ The crystal surfaces constructed are not perfectly flat but have their roughness with the corresponding rugosity values being between 1.3 and 1.8 in this study which are typical of surface tapping mode AFM rugosity values reported in the literature.^36−38^ The virtual probe used is below the size of the typical AFM probe contact surface areas which are typically in the range of 10^–9^ m^–2^, and scanned over substrate surface areas of 1.5 μm × 1.5 μm to 10 μm × 10 μm.^36,37,39,40^ The modeling actually is very able to probe contact energies within the scale of surface roughness of most pharmaceuticals, and indeed within the scale of single asperity contact surface area reported for AFM colloid probe tips for micronized inhalation compounds.^40^

The high stresses during mixing/blending might result in surface defects of particles and/or a transition to a partially disordered state, which can make the computational and experimental studies much more complicated. Application of mechanical force creates surface disorder and amorphous content and reveals higher-energy crystal facets that typically contribute little to the surface area of the crystallized particle.^41,42^ The methods developed in this study will be applicable with clearer molecular definitions of these sites of surface disorder surfaces. It is also worth noting that the existence of “active sites” on carrier lactose surfaces may comprise high-surface-energy sites and/or regions of macro-rugosity to which drug particles become tightly bound. There is convincing evidence that such active sites become saturated at low drug loadings.^43,44^ At higher loading concentrations of fine particles (drug and excipient fines) typical of ternary DPI formulations, the study of adhesion forces to all crystal facets relevant beyond those “active sites” is highly relevant. In brief, the computational approach reported here enables prediction of the force of adhesion for contact of particles at the level of microscale surface rugosity, and for active sites at the scale of particle contact area to high-energy facets and within macroscale rugosity surface clefts.

Here, the intermolecular and surface–molecule searches between TBS and LMH, and also between two most important surfaces from TBS ({001}, {010}) and LMH ({010}, {0-11}), as identified by the simulation results from the intermolecular and molecule–surface systematic grid searches, were investigated. The grid search simulations carried out are listed in Table 1. In practical particle manufacturing and formulation processes, two interactive surfaces will not always have the same area and even if they do, they may not fully face each other. Therefore, in realistic cases, the two interactive faces will have different surface areas. To mimic this, the interactions between different individual surfaces were simulated by the combinations of each face acting as a slab surface and then a probe surface (e.g., the two runs of the consecutive two rows in the TBS–LMH column of Table 1).

**Table 1:** List of Surface–Surface Searches for Two TBS Faces ({001}, {010}) and Two LMH Faces ({010}, {0–11})

TBS–TBS	TBS–LMH	LMH–LMH
TBS{001} – TBS{001}	TBS{001} – LMH{010}	LMH{010} – LMH{010}
TBS{010} – TBS{010}	LMH{010} – TBS{001}	LMH{0–11} – LMH{0–11}
TBS{001} – TBS{010}	TBS{001} – LMH{0–11}	LMH{010} – LMH{0–11}
TBS{010} – TBS{001}	LMH{0–11} – TBS{001}	LMH{0–11} – LMH{010}
	TBS{010} – LMH{010}	
	LMH{010} – TBS{010}	
	TBS{010} – LMH{0–11}	
	LMH{0–11} – TBS{010}	

### 2.3 Experimental Investigation

#### 2.3.1 Preparation of Model Inhalation Blends

Realistic inhalation blends were prepared using the sieved lactose monohydrate carrier Lactohale 100 (LH100) along with micronized terbutaline sulfate and micronized lactose monohydrate Lactohale 300 (LH300). Lactohale powders were donated by DFE Pharma (Germany), while micronized terbutaline sulfate was provided by AstraZeneca (Sweden).

Our previous work revealed the importance of disrupting large drug agglomerates in the feedstock of micronized API,^18^ and for this reason, blends were prepared using a Hosokawa High Shear mixer with the Picomix module (Hosokawa Micron Ltd., Runcorn, U.K.). During the blending processes, a constant rotational speed of 1000 rpm and a total mixing time of 2 min were used^45^ to keep the input mixing energy constant for all of the prepared powders.^46^ To produce the blends with additional micronized components TBS or LH300 particles (10% w/w), the Lactohale 100 carrier was first mixed at 1000 rpm for 1 min to achieve more uniform distribution of the LH100 powder. Then, the appropriate mass of fines was added into the mixer for a further 1 min of mixing at 1000 rpm to prepare TBS-LH100 and LH300-LH100 blends, respectively. For control, the LH100 powder was also blended for an equivalent protocol.

#### 2.3.2 Microstructural XCT Characterization of the Inhalation Blends

A qualitative evaluation of the 3D blend microstructure was performed by XCT. Two blended powder samples were prepared comprising LH100 with 10% w/w LH300 and LH100 with 10% w/w μTBS. These were filled into 2 mm diameter Kapton tube sample holders^47^ and scanned using a Zeiss Xradia Versa 520 X-ray microscope using the following settings: voltage 40 kV, power 3 W, source–sample distance 9.0 mm, and sample–detector distance 8.5 mm. A 20× objective and 2× camera binning were used to acquire 1601 projections with an exposure of 12.5 s per projection for each scan. The projections were reconstructed using the native Zeiss reconstruction software to generate a virtual volume (tomogram).^48,49^ Following Gajjar et al (2023),^26^ the virtual volume was segmented into separate drug/micronized extrinsic fines (μTBS/LH300) and carrier lactose phases. Visualizations in 2D and 3D were produced using Dragonfly Pro 4.1 (Object Research Systems, Canada).

#### 2.3.3 Functional Assessment of Microparticle Cohesion and Adhesion in Blends

Laser diffraction analysis was performed to assess the cohesive behavior of the micronized particles when formulated as a powder blend with the inhalation carrier-grade lactose LH100, by monitoring the change in particle size distribution (PSD) and the volume fraction below 4.5 μm. The percentage below 4.5 μm was used as a measure of redispersion of the micron-sized component from the blends. Particle size measurements were performed on a HELOS/RODOS Laser Diffraction unit, equipped with the ASPIROS dispersing system (dispersing aperture diameter 4 mm, feed velocity 25 mm/s) (Sympatec GmbH, Clausthal-Zellerfel, Germany). The R5 lens was used for the blends and the R2 lens for micronized TBS and LH300 raw materials. Powder was filled into the ASPIROS glass vials and dispersed via vacuum suction at 0.2 and at 2.0 bar. Particle size distributions (PSDs, triplicate samples) were calculated using the Fraunhofer theory and were analyzed using the WINDOX 5.3.1.0 software.^50^ Fraunhofer theory was used for analysis of scattering patterns to determine PSDs to avoid inappropriate choices^51^ of real and imaginary refractive indices which would be required to employ Mie scattering theory.^52^ The current analysis generated scattering patterns for mixed aerosol clouds of different particle chemistries (i.e., TBS, LMH) and physical states of crystal size (i.e., micronized and carrier) and degrees of agglomerations (i.e., μTBS and μLMH) that depend on dispersion airflow pressure. As such, an accurate estimate of both real and imaginary refractive indices of the particle clouds would not be possible, and the Fraunhofer approximation was chosen to maintain consistency of modeling assumptions for comparability between different blends and dispersion pressures.

## 3 Results and Discussion

### 3.1 Prediction of Cohesive and Adhesive Energies

#### 3.1.1 Molecule–Molecule Interaction Analysis

**Figure 3:**
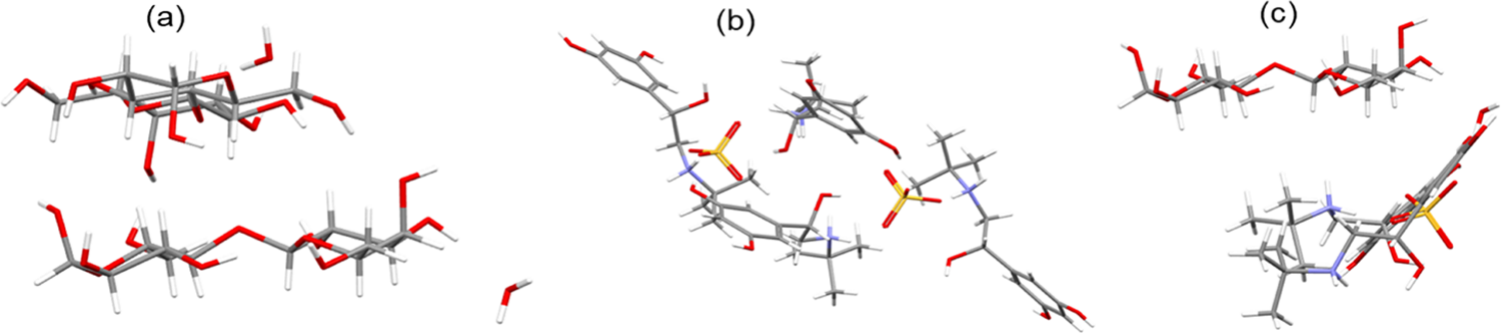
Lowest-energy (preferential) intermolecular binding structures for (a) LMH–LMH molecules, (b) TBS–TBS molecules, and (c) TBS–LMH molecules.

**Figure 4:**
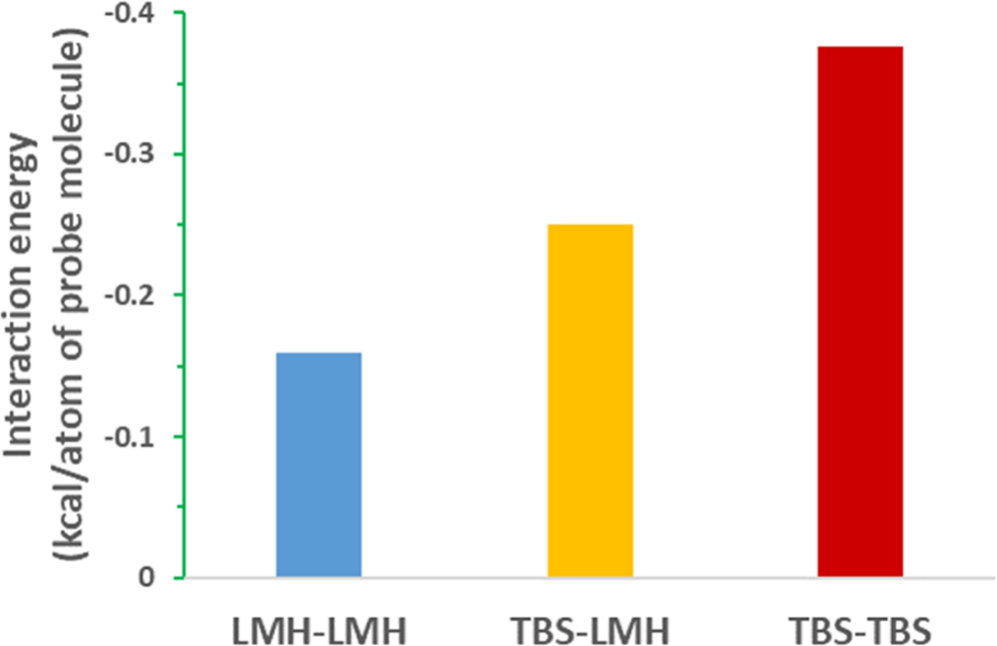
Interactions (binding) energy between LMH–LMH, TBS–LMH, and TBS–TBS molecules (note that 1 kcal = 4.184 kJ).

An example of the grid search surrounding the LMH molecule and the best binding position (lowest interaction energy) between the LMH–LMH, TBS–LMH, and TBS–TBS molecules is shown in Figure 3. The interaction (binding) energy between LMH–LMH, TBS–LMH, and TBS–TBS molecules are shown in Figure 4, with the TBS–TBS interactions being the strongest with highest cohesive tendency, followed by the TBS–LMH interactions with the LMH–LMH interactions being the weakest.

#### 3.1.2 Molecule–Surface Interaction Analysis

**Figure 5:**
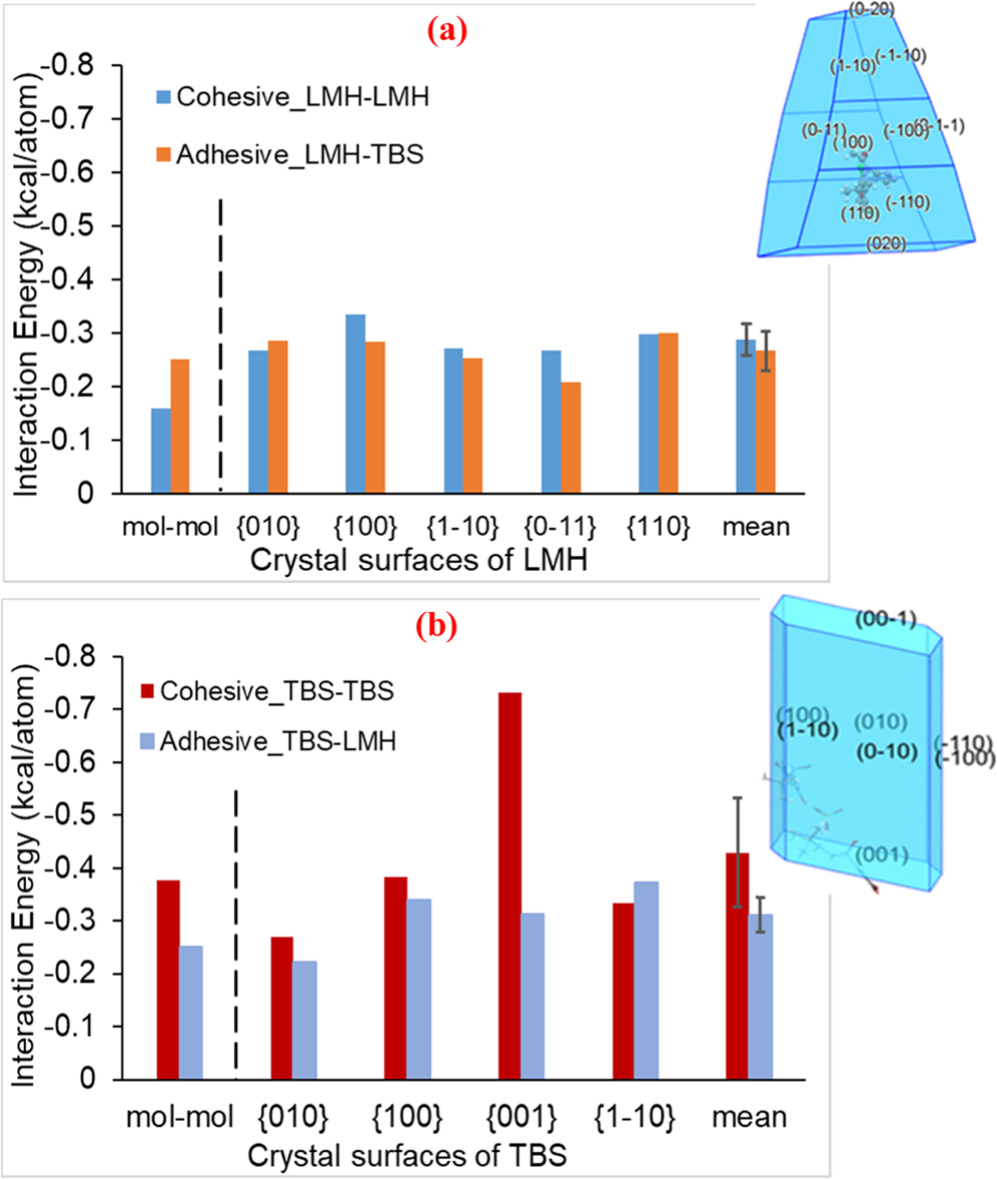
Comparative individual interaction (binding) energies together with the means and standard deviations (error bar) (a) between LMH and LMH molecules, LMH molecule on LMH crystal surfaces (cohesive), and TBS molecule on LMH crystal surfaces (adhesive) and (b) TBS and TBS molecules, TBS molecule on TBS crystal surfaces (cohesive), and LMH molecule on TBS crystal surfaces (adhesive). Note that 1 kcal = 4.184 kJ.

The interaction energies between various molecule–crystal surface combinations of TBS and LMH are summarized in Figure 5. Note that the crystal morphologies were predicted based upon a selection of the most stable crystal surfaces as identified by the BFDH approach^53−201^ which lower the morphological importance of some surfaces, for example, the stable surface for {010} growth would be {020}.

The interaction (binding) energies decrease in the order TBS–TBS > TBS–LMH> LMH–LMH > LMH–TBS except for interactions on the LMH {010} face and the TBS {1–10} face. This is consistent with the calculation of the molecule–molecule interactions. Also, the cohesive strengths for LMH on all of the LMH surfaces were found to be very similar with the lowest standard deviation of 0.026 kcal/atom (0.109 kJ/atom) for the mean interaction force (see further detail in the SI (Table S1)). However, the interaction energies for TBS–TBS faces were found to be dependent on the TBS surfaces examined with the TBS {001} having about 2 times of cohesive strength compared to the other 3 TBS surfaces. While the TBS–LMH adhesive strength was slightly higher than the LMH–TBS adhesive strength, the mean binding energies were found to be very similar to the calculated LMH–LMH cohesive strengths with both being lower than the TBS–TBS cohesive strength. Examination of the mean interaction forces from the modeling predicts likely blend segregation, with TBS–TBS cohesion being strongly favored compared to TBS–LMH adhesion, and LMH–LMH cohesion and approximately equal to TBS–LMH adhesion.

#### 3.1.3 Surface–Surface Interaction Analysis

**Figure 6:**
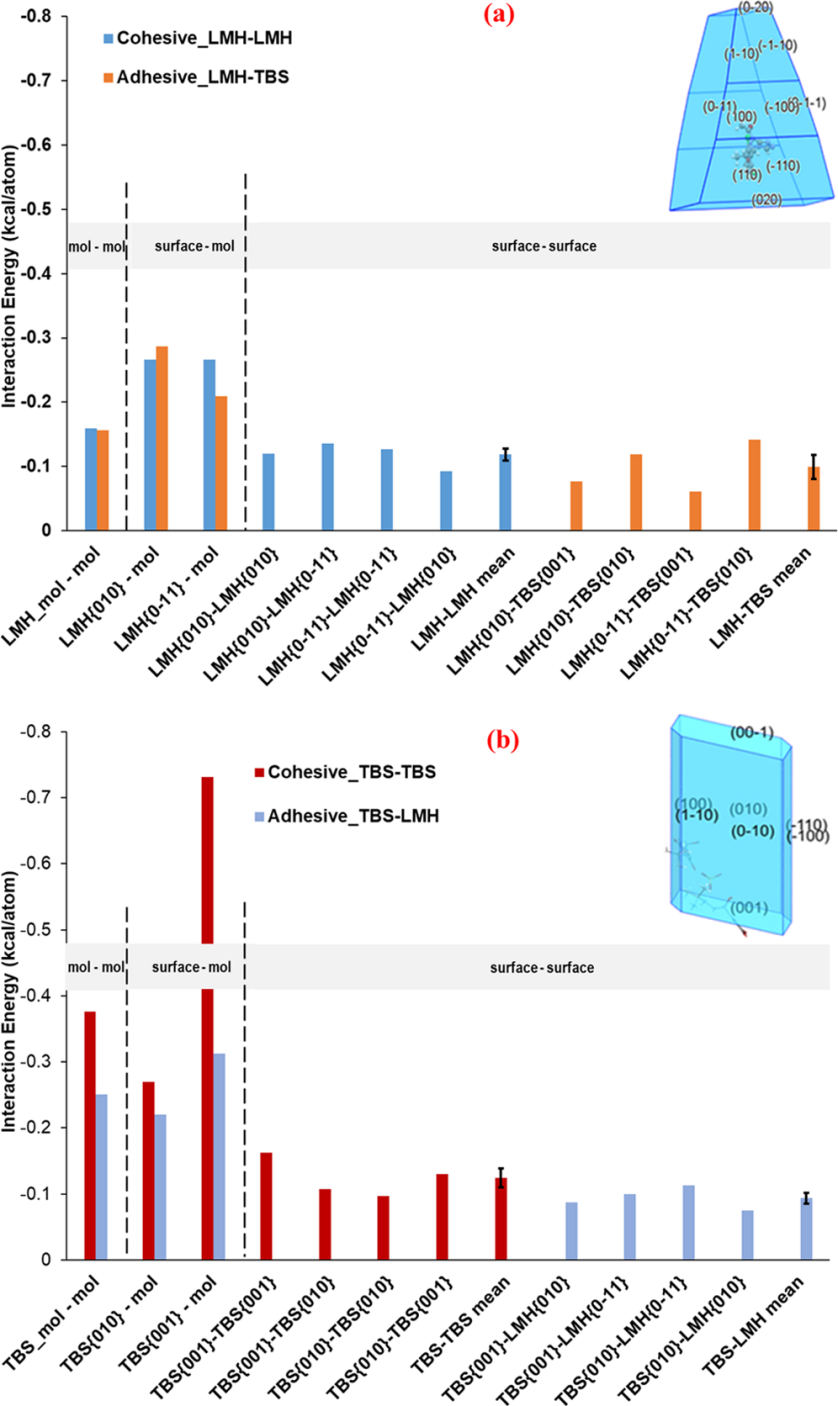
(a) Predicted interaction energies between LMH and LMH, between LMH and TBS, the interaction (binding) energies of LMH on the LMH and TBS on the LMH crystal surfaces for the cohesive strengths of LMH surface and LMH molecule, and LMH surface and TBS molecule, and also the cohesive strengths of LMH surfaces–LMH surfaces and the adhesive strengths of LMH surfaces–TBS surfaces. (b) Molecular interaction energy between TBS and TBS, and TBS and LMH, the interaction (binding) energies of TBS on the TBS and LMH on the TBS crystal surfaces for the cohesive strengths of TBS surface and TBS molecule, and TBS surface and LMH molecule, and also the cohesive strengths of TBS surfaces–TBS surfaces and the adhesive strengths of TBS surfaces–LMH surfaces. Note that 1 kcal = 4.184 kJ.

Figure 6 shows the intermolecular grid-search results highlighting the comparison between the calculated interaction energies between molecule and molecule, between molecule and crystal surface, and between two crystal surfaces. The mean energies from the surface–surface search results based on LMH{010}, LMH{0–11}, TBS{001}, and TBS{010} as the host are also plotted with the corresponding standard deviations being shown as error bars. These crystal surfaces were selected reflecting their possessing the highest contribution to the surface area of the crystals. Overall, as shown in Figure 6a and Table S1, the cohesive energies between LMH crystal surfaces have a mean value of −0.119 kcal/atom (−0.498 kJ/atom) and a standard deviation of 0.018 kcal/atom (0.075 kJ/atom) which is less than half of those for the LMH–LMH surface–molecule searches (−0.288 kcal/atom (−1.205 kJ/atom) and 0.026 kcal/atom (0.109 kJ/atom)). It is clear from Figure 6 that, the results from the surface–surface and surface–molecule searches (Figure 5) are well aligned with the TBS–TBS cohesive energies being the strongest. The mean value for the TBS–TBS cohesive energies is significantly higher than other interactions with the strongest interaction being TBS{001}-TBS{001} at −0.162 kcal/atom (−0.678 kJ/atom) (details of all interactions are presented in the SI (Table S1)). The surface–surface searches also show that LMH–LMH cohesive interactions were the second strongest and hence the order of the interaction energies decreased in a slightly different order with respect to the surface–molecule searches: TBS–TBS > LMH–LMH > LMH–TBS > TBS–LMH. These findings indicate once more the prediction of a cohesively balanced powder blend, in which blend segregation of TBS from the LMH diluent would be expected to occur.

The means of the total interaction, dispersive and polar energies of LMH–LMH and TBS–LMH (see further details in the SI (Figures S1a,d)) are similar with their corresponding differences of −0.025, −0.01, and −0.014 kcal/atom (−0.105, −0.042, and −0.059 kJ/atom), respectively, and small in comparison to TBS–TBS and LMH–TBS interactions. This, together with the corresponding standard deviations being also small and similar, demonstrates the consistent trend for all three searches (molecule–molecule, surface–molecule, and surface–surface). It also indicates that the interactions of LMH–LMH, TBS–LMH, and LMH–TBS are weaker than TBS–TBS. However, for the interactions of TBS–TBS and LMH–TBS (further details in the SI (Figure S1c,b)), these contributions are dominated by the polar energy which becomes the dominant contribution to the total interaction energy, particularly for TBS–TBS. Further splitting the polar energy into the hydrogen bonding and electrostatic contributions (Figure S2) shows that the dispersive and hydrogen bonding energies dominate the total energies for the interaction between LMH–LMH (Figure S2a). For the LMH–TBS interactions (Figure S2b), the hydrogen bonding and electrostatic interactions have a similar effect on the total energies. The electrostatic contributions play a more important role than the hydrogen bonding to the total energies (Figure S2b,c), particularly for the interactions between TBS–TBS (Figure S2c).

The full energy distributions of molecule–surface and surface–surface intermolecular interactions, together with their mean energies and standard deviations from Gaussian fittings, can be found in the Supplementary Information (SI) (Figure S3 and Table S2).

### 3.2 Qualitative Experimental Assessment of Cohesive-Adhesive Tendencies

#### 3.2.1 Characterization of Blend Microstructure

**Figure 7:**
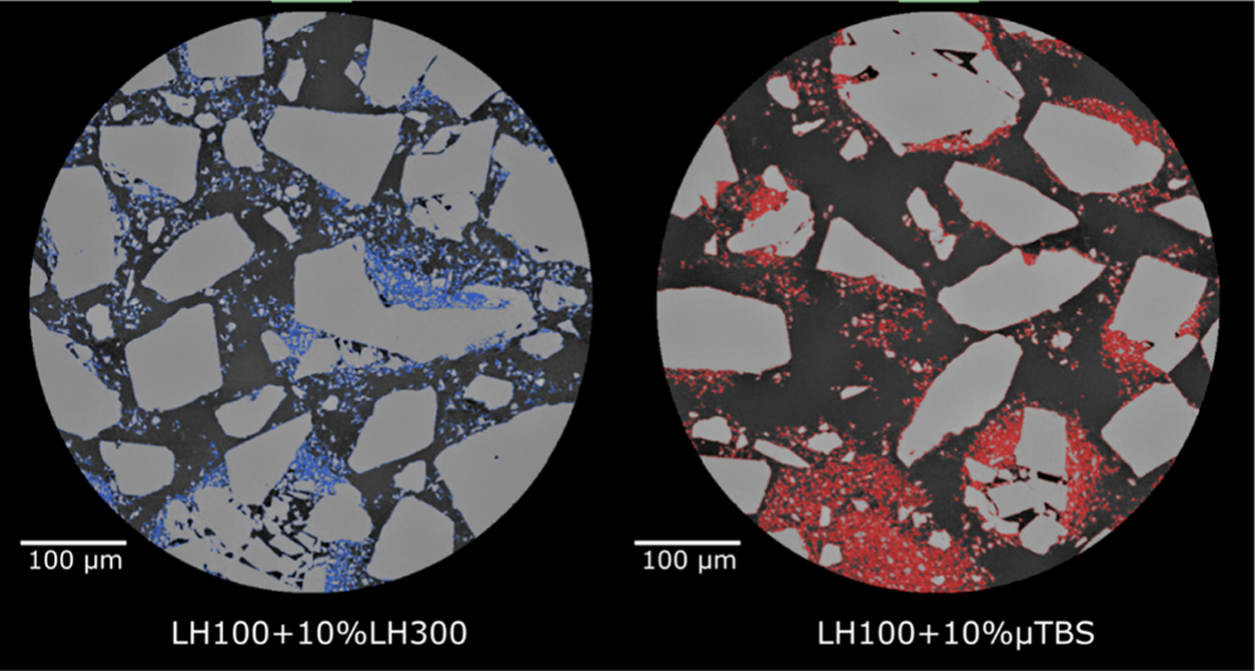
Virtual horizontal XCT slices through 10% w/w blends of LH100 with LH300 (left, rendered blue) and μTBS (right, rendered red). Light gray is the lactose phase, while the darkest grayscale value is the air.

Figure 7 compares horizontal virtual 2D XCT renderings through the two prepared blends. It can be seen that LH300-rich regions are small and dispersed across the cross section, with no particular or preferential binding to specific facets of the carrier lactose particles, or as a distinct subphase in the voids between carrier particles. On the other hand, the μTBS is concentrated in small pockets close to each other, with poor association with the carrier particle surfaces. This is shown by the speckled red regions close together, with very small air pockets (noncolored regions) in between.

**Figure 8:**
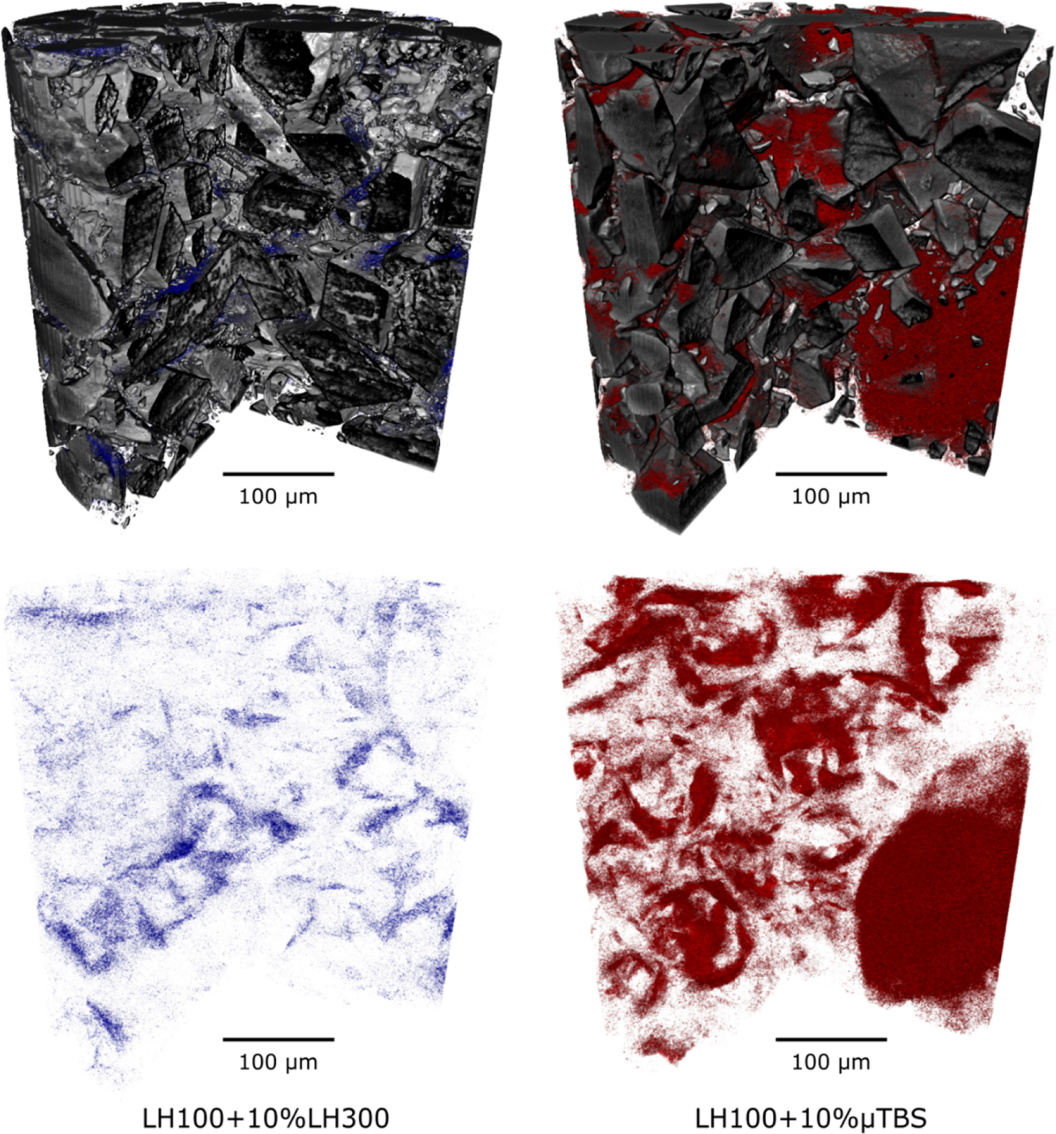
3D renderings of the XCT data for 10% w/w blends of LH100 with LH300 (left) and μTBS (right). The upper tile shows both the lactose (gray) and drug phases (color), while the lower tile shows only the drug phase (lactose phase is transparent).

This differential behavior between the two materials (LH300 and μTBS) can be seen in 3D in Figure 8, with the LH300-LH100 blend containing very small LH300-rich regions that are dispersed relatively uniformly throughout the blend, while the μTBS-LH100 blend displayed is more highly segregated with localized drug-rich regions. In particular, a very large drug-rich region can be seen in the lower-right portion of the sample. Visually, the carrier lactose surfaces also have thicker μTBS-rich regions in contact with them, which may be due to the larger size of agglomerates that adhere to the lactose particle surfaces, and which may have not been adequately disrupted despite the high-shear blending operation.

These qualitative blend microstructures are consistent with the molecular modeling results. The localized μTBS-rich regions result from the cohesive TBS–TBS interactions having higher energy than TBS–LMH or LMH–LMH interactions, thus leading to blend segregation where large cohesive masses of drug agglomerates have been dispersed between carrier particles, rather than individual drug particles or small agglomerates coating the carrier particles uniformly. Consequently, the TBS blend exhibits low porosity with dense regions of agglomerates occupying the interparticulate voids. In the case of LH300-LH100 blends, the interactions are adhesively balanced and so the LH300 microparticles are diffusely dispersed throughout the blend, leaving the blend with high air permeability. The molecular modeling also revealed that the highest forces of TBS–TBS slab–slab cohesion are associated with the (010) and (001) crystal surfaces, which contribute the majority of the surface area of the TBS crystalline particles. The TBS_(001)_ and TBS_(010)_ cohesive forces are also larger than the TBS_(010)_-LMH_(010)_, TBS_(010)_-LMH_(011)_, TBS_(001)_-LMH_(010)_, and TBS_(001)_-LMH_(011)_ adhesive forces, where {010} and {011} are the dominant faces on the LMH particle surface area. The LMH–LMH cohesive forces are more evenly balanced across all crystal facet interactions, and as a result, LH300 particles can adhere homogeneously around the LH100 particle surface, with lower sensitivity to the geometry of particle–surface contacts than for TBS–LMH interactions. Note that the direct comparisons between predicted and measured surface energies of TBS^17^ and LMH^18,22^ have been reported in the literature with good agreement. In this study, the qualitative comparisons between experimental observations and molecular modeling simulations provided an in-depth explanation of the blend behavior at a microscale level, hence useful guidance for designing and enhancing practical particle blend processes.

#### 3.2.2 Characterization of Powder Blend Physical Properties

**Figure 9:**
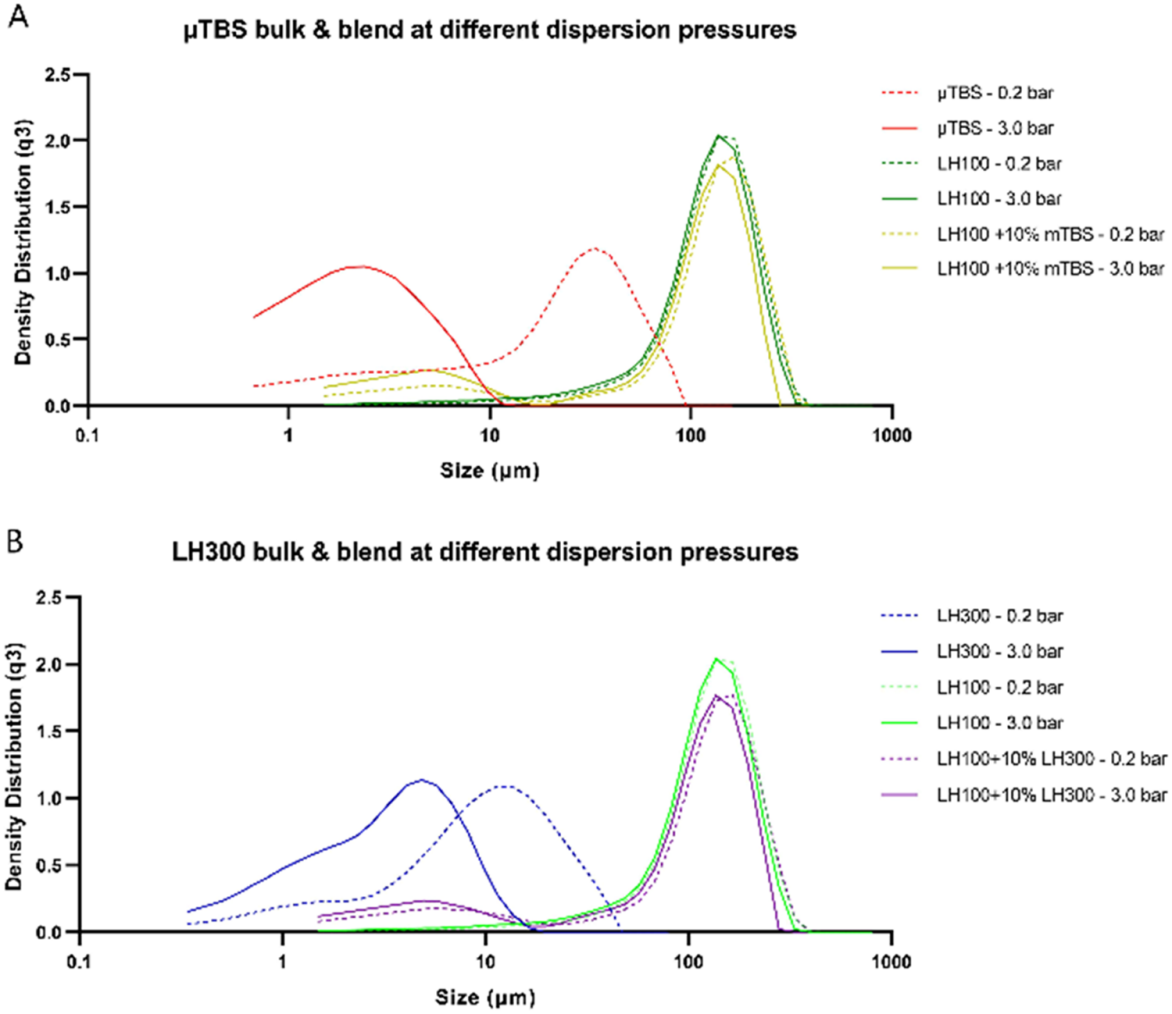
Representative PSDs at 0.2 and 3.0 bar dispersion pressures of (A) micronized TBS (μTBS), Lactohale 100, and Lactohale 100 blended with 10% w/w micronized TBS (μTBS) and (B) Lactohale 300, Lactohale 100, and Lactose 100 blended with 10% w/w Lactohale 300.

Analysis of the particle size measurements, as shown in Figure 9, demonstrates that the LH300-LH100 and μTBS-LH100 blends possess a wider particle size distribution (PSD) than any of the raw materials on their own. It can be seen from the PSDs that the respective powder blends of 10% fines with LH100 carrier lactose possessed a wider distribution than the component raw materials at both 0.2 and 3.0 bar dispersion pressures. The highly cohesive behavior of μTBS (Figure 9A) prior to high-shear mixing is evident from mode of the distribution at ∼50 μm in size when dispersed at 0.2 bar, despite the individual particles being substantially smaller than this (as evidenced by the shift in the mode of the PSD to lower sizes when dispersed at 3.0 bar). This contrasts with micronized lactose (LH300, Figure 9B), which dispersed readily at pressures as low as 0.2 bar, and for which there are minimal changes to the modal size when the dispersing pressure was increased to 3.0 bar. A shoulder between 10 and 100 μm on the LH100 principal mode is more prominent at 0.2 bar for μTBS-LH100 blends than for LH300-LH100 blends, consistent with a population of large TBS agglomerates, that had failed to be disrupted during blending.

**Figure 10:**
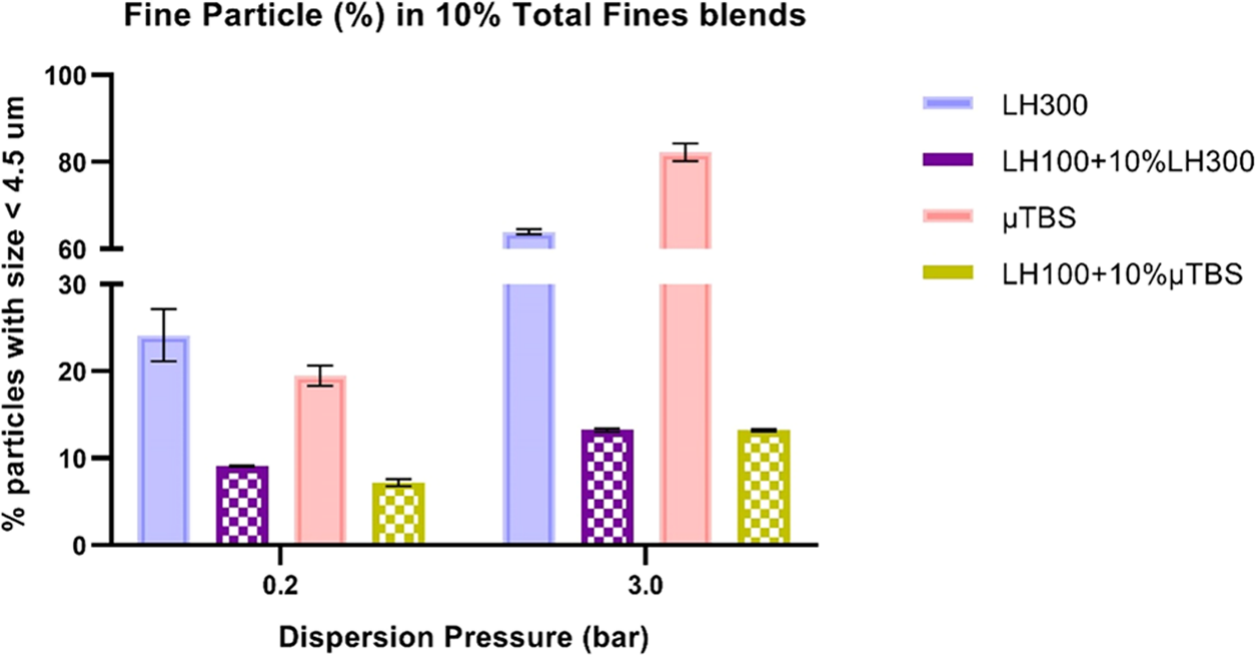
Summary of laser diffraction PSD data highlighting the fine particle fraction (percentage less than 4.5 μm) released from the micronized terbutaline sulfate (μTBS), micronized lactose (LH300), and blends of μTBS and LH300, respectively, with carrier-grade lactose (LH100) at 0.2 and 3.0 bar dispersion pressures (mean ± SD, *n* = 3 determinations).

The percentage of particles less than 4.5 μm (i.e., the respirable fraction), was statistically significantly lower for μTBS-LH100 blend compared to LH300-LH100 blend at 0.2 bar dispersion pressure (Figure 10) despite the smaller size of individual μTBS micronized particles compared to LH300 particles. The percentage less than 4.5 μm was higher for both LH300-LH100 and μTBS-LH100 blends at 3.0 bar dispersion pressure compared to 0.2 bar; however, there was no significant difference between μTBS and LH300 blends at 3.0 bar. This indicated the requirement for a very high air pressure to disperse the agglomerated phase of TBS within the blends, consistent with the substantial increase in the percentage less than 4.5 μm for the raw TBS material as well. Therefore, the blend segregation for μTBS seen in the XCT was also evident from the particle size characterization and associated with functional differences in the dispersion of μTBS from an LH100 blend compared to the LH300 blend. The latter was consistent with an adhesive behavior of LH300 to the larger LH100 carrier lactose particles.

### 3.3 Discussion

The behavior of inhalation formulations is particularly dominated by colloidal forces of surface–surface interactions.^56^ Consequently, inhalable API particles are typically cohesive and agglomerative as bulk materials, but when formulated with excipients demonstrate a spectrum of behavior from adhesion (i.e., interact with excipient substances) to cohesion (i.e., remain segregated as agglomerates within the formulation), which dominates blending, flow, aerosolization, and drug delivery performance. Colloid probe microscopy and surface energy analysis have emerged to characterize the balance of cohesive and adhesive forces between the components of inhalation formulations.^36,57,58^ Both techniques have provided a powerful ability to predict and engineer formulation behavior.^59,60^ However, the colloid probe microscopy approach is challenging to undertake, time-consuming, and limited to the exploration of a small number of particles. It is also difficult to identify the actual crystal facet being studied on the colloid probe. In the case of surface energy analysis, relatively large amounts of material are required to produce the data, and there has been inconsistency in the ability of surface energy data to predict formulation performance.^61^

The interactions between particles that govern formulation behavior and performance are defined by the intermolecular forces at the particle surface. These electrostatic, dispersive (and potentially capillary) forces are determined by the crystallography of the individual crystal facets that are in contact within the formulation. Molecular modeling techniques are now widely applicable for understanding and controlling crystallization processes to design optimum properties into the final particles (Turner et al.,^13^ Wang et al., 2021,^14^ Moldovan et al.^15^), as well as understanding such properties as crystal interface stability,^34^ and face-specific wetting by dissolution solvents.^19^ As part of the transition to Industry 4.0, the development of computational tools for predictive interpretation of formulation design, manufacture, stability, and performance are key to developing robust digital twins in future for finished pharmaceutical products.

Inhalation formulations represent a particular challenge for both quality-by-design and performance-engineering of formulated products using the materials science tetrahedron approach.^5^ Due to the small particle sizes (and high specific surface areas) required for API deposition within the lung airways (1–5 μm) the consequent high density of small particles within the formulation imposes incredible difficulty in characterizing the physical structure of formulations. Indeed, to date, attempts to characterize dry powder inhalation blend structure have relied upon tests that are incapable of examining the bulk formulation since they require destructive sampling of the powder (e.g., combinations of electron microscopy, morphologically directed Raman imaging, dissolution, or single-particle aerosol mass spectrometry^62−64^). Recently, we developed a nondestructive XCT approach for microstructural imaging of DPI powder blends prior to aerosolization.^65^

Previous work using the synthonic engineering approach to predict inhaled formulation behavior employed molecular probes on computer-simulated crystal surfaces did not successfully predict the blending of another API, fluticasone propionate, with LMH carrier particles.^18^ In designing the current study, we therefore developed a slab–slab modeling approach for prediction alongside the use of high-shear blending for formulation manufacture to facilitate intimate surface–surface contact of drug particles that enter the formulation in agglomerated structures. The high-shear blending protocol was designed to provide the best opportunity for agglomerate dispersal and interaction with the carrier particle surfaces.^66^ The combination of modeling and XCT imaging was demonstrated in the current study to successfully predict the behavior of the micronized materials (TBS and LH300) with a typical DPI carrier-grade lactose (LH100). The *in silico* modeling of “slabs” of crystalline material representing different crystal faces successfully predicted the blend segregation of μTBS and its persistence as a highly agglomerated subphase within the powder bed. Furthermore, this segregation was also observed to translate through to functional differences in the aerosolization behavior compared to a blend of micronized lactose (LH300) with the same carrier. The segregation of TBS from, and the adhesion of micronized lactose to, LMH carriers agrees with the findings of several studies.^46,67−69^ The crystal surface dependency of cohesive/adhesive balance predicted in the current work for TBS and LMH is supported by the finding that spray-dried TBS produced greater liberation of fine particles from LMH blends compared to micronized crystals of TBS.^69^ This confirmed that the surface–surface modeling developed in the current study represents a major advance for predictive pharmaceutics. Furthermore, the combination of *in silico* modeling with XCT provides a powerful integrated workflow for the further development of digital twins of inhaled formulations.

## 4 Concluding Remarks

In this study, grid-based systematic search methods were used to investigate the interparticle interactions between molecule and molecule, molecule and surface, and surface and surface. Terbutaline sulfate as an API and α-lactose monohydrate as an excipient were investigated as a model system. It was found that specific crystal faces can directly affect particle behavior during product formulation and delivery processes through the different cohesive and adhesive interaction energies of the two compounds. The modeling results demonstrate that the cohesive interactions of TBS–TBS are much stronger than the adhesive interactions between TBS and LMH, and also the cohesive interaction of LMH–LMH. This is in good qualitative agreement with the powder blend PSD measurements and 3-D XCT studies.

These simulations highlight the applicability of the methods to guide the formulation design of such inhalation powders, in order to achieve optimum aerosolization and efficacy. In silico characterization as part of a digital design strategy can inform formulation development for inhaled medicines to maintain effective drug aerosolization in delivery devices. From this, the adhesive and cohesive interactions between API and excipient are characterized. The utility of such molecular modeling approaches as part of a digital design strategy for inhaled medicines exhibits benefits for inhaled medicine R&D.

Further work will include multiple particle interactions of TBS–TBS, LMH–LMH, and TBS–LMH using systematic search methods, hence further mimicking the interactions in more practical formulation processes.

## Supporting Information Available

Total interaction, dispersive and polar (hydrogen bonding and electrostatic) energies (Figures S1 and S2, Table S1) between molecules, surface and molecule, and surface and surface of LMH–LMH, LMH–TBS, TBS–TBS and TBS–LMH, and their distributions with the fitted curves using a Gaussian function (Figure S3 and Table S2) (PDF)

## Abbreviations

a, b, c: crystal unit cell parameters (Å)
α, β, γ: crystal unit cell parameters (°)
μTBS: micro size of TBS
API: active pharmaceutical ingredient
*d*_*hkl*_: thickness of the growth step layer (Å)
Dv50: median particle size by volume
(*h k l*): miller index
IGC: inverse gas chromatography
LH: lactohale
LMH: lactose monohydrate (α-form)
PSD: particle size distribution
QbD: quality by design
TBS: terbutaline sulfate
XCT: X-ray computed tomography
