# Supplementary material for: Predicting the Strength of Cohesive and Adhesive Interparticle Interactions for Dry Powder Inhalation Blends of Terbutaline Sulfate with α-Lactose Monohydrate
Source: Mol Pharm. 2023 Sep 8;20(10):5019–31. doi: 10.1021/acs.molpharmaceut.3c00292 (PMC10548469; doi:10.1021/acs.molpharmaceut.3c00292)
Supplement: Supplementary file 1 — mp3c00292_si_001.pdf [file mp3c00292_si_001.pdf]

Supporting Information

Predicting the Strength of Cohesive and Adhesive Inter-Particle Interactions for Dry Powder Inhalation Blends of Terbutaline Sulphate with α-Lactose Monohydrate

Cai Y. Ma[1], Thai T. H. Nguyen[1,$], Parmesh Gajjar[2,§], Ioanna D. Styliari[3], Robert B. Hammond[1], Philip J. Withers[2], Darragh Murnane[3,*], Kevin J. Roberts[1,*]

[1] Centre for the Digital Design of Drug Products, School of Chemical and Process Engineering, University of Leeds, Leeds, LS2 9JT, UK

[2] Henry Royce Institute, School of Materials, University of Manchester, Oxford Road, Manchester, M13 9PL, UK

[3] School of Life and Medical Sciences, University of Hertfordshire, College Lane, Hatfield, AL10 9AB, UK

Current addresses:

[$] School of Computing, University of Leeds, Leeds, LS2 9JT, UK

[§] Seda Pharmaceutical Development Services, Unit D Oakfield Road, Cheadle Royal Business Park, Cheadle, SK8 3GX, UK

[*] Corresponding authors: k.j.roberts@leeds.ac.uk; d.murnane@herts.ac.uk

This supplementary information supports the main manuscript by providing further details of the followings: **Figure S1** shows the total interaction, dispersive and polar energies between molecules, surface and molecule, and surface and surface of LMH – LMH, LMH – TBS, TBS – TBS and TBS – LMH, with the mean energies and standard deviations for the four groups. By further separating the polar into hydrogen bonding and electrostatic energies, **Figure S2** shows the total interaction, dispersive, hydrogen bonding and electrostatic energies between molecules, surface and molecule, and surface and surface of LMH – LMH, LMH – TBS, TBS – TBS and TBS – LMH, with the mean energies and standard deviations for the four groups. **Figure S3** shows the interaction (binding) energy distributions of surface-molecule and surface-surface searches including the fitted curves using a Gaussian function. The means and standard deviations of the Gaussian fittings are listed in **Table S2**. **Table S1** shows the systematic search results for interaction energies of TBS molecule – LMH molecule, TBS surface – LMH molecule and LMH surface – TBS molecule, TBS faces ({001}, {010}) - LMH faces ({010}, {0-11}). **Table S2** presents the means and standard deviations of interaction energy distributions after Gaussian fittings as shown in **Figure S3**.

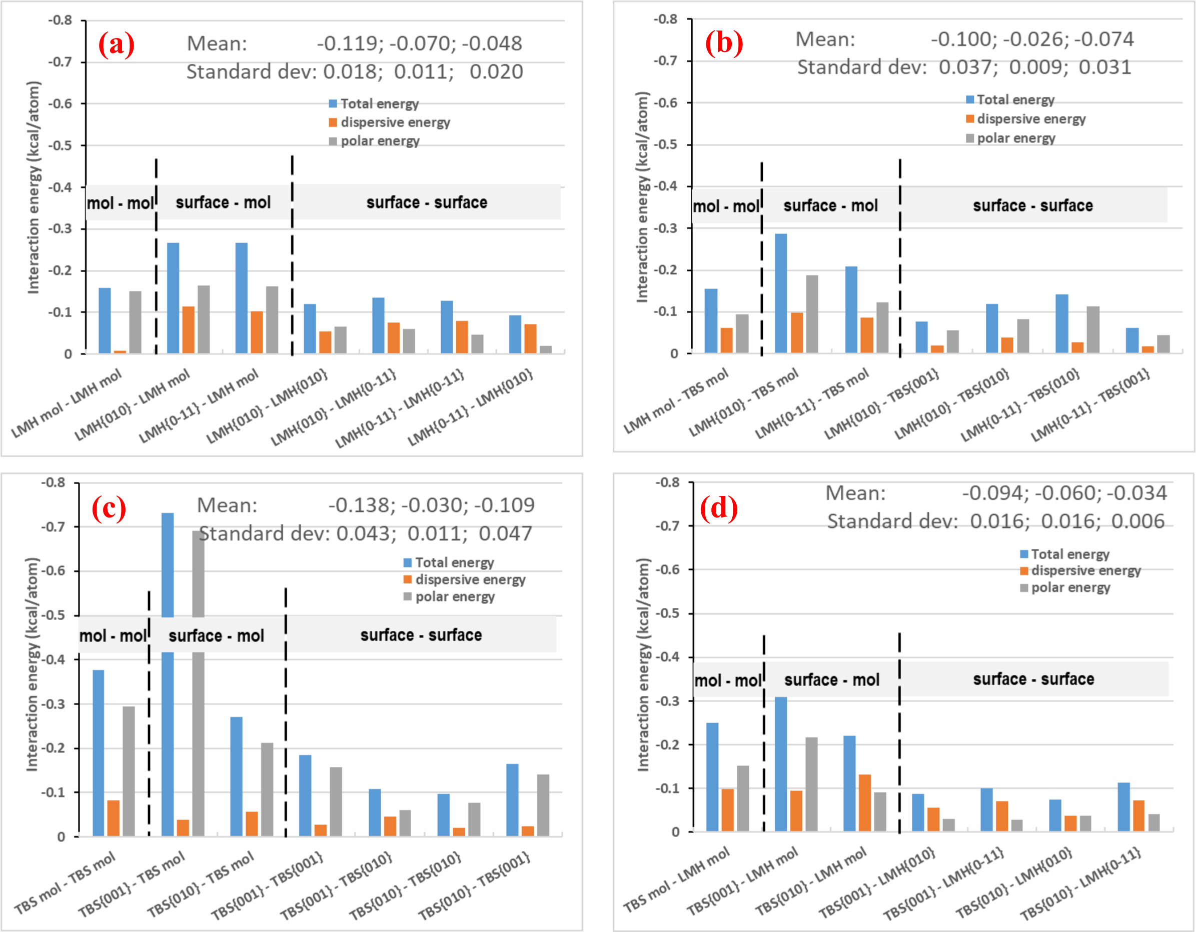

Figure S1. Total interaction, dispersive and polar energies of molecule and molecule, surface and molecule, and surface and surface of (a) LMH – LMH, (b) LMH – TBS; (c) TBS – TBS, and (d) TBS – LMH, with the mean energy and standard deviation for the four groups. Note that 1 kcal = 4.184 kJ.

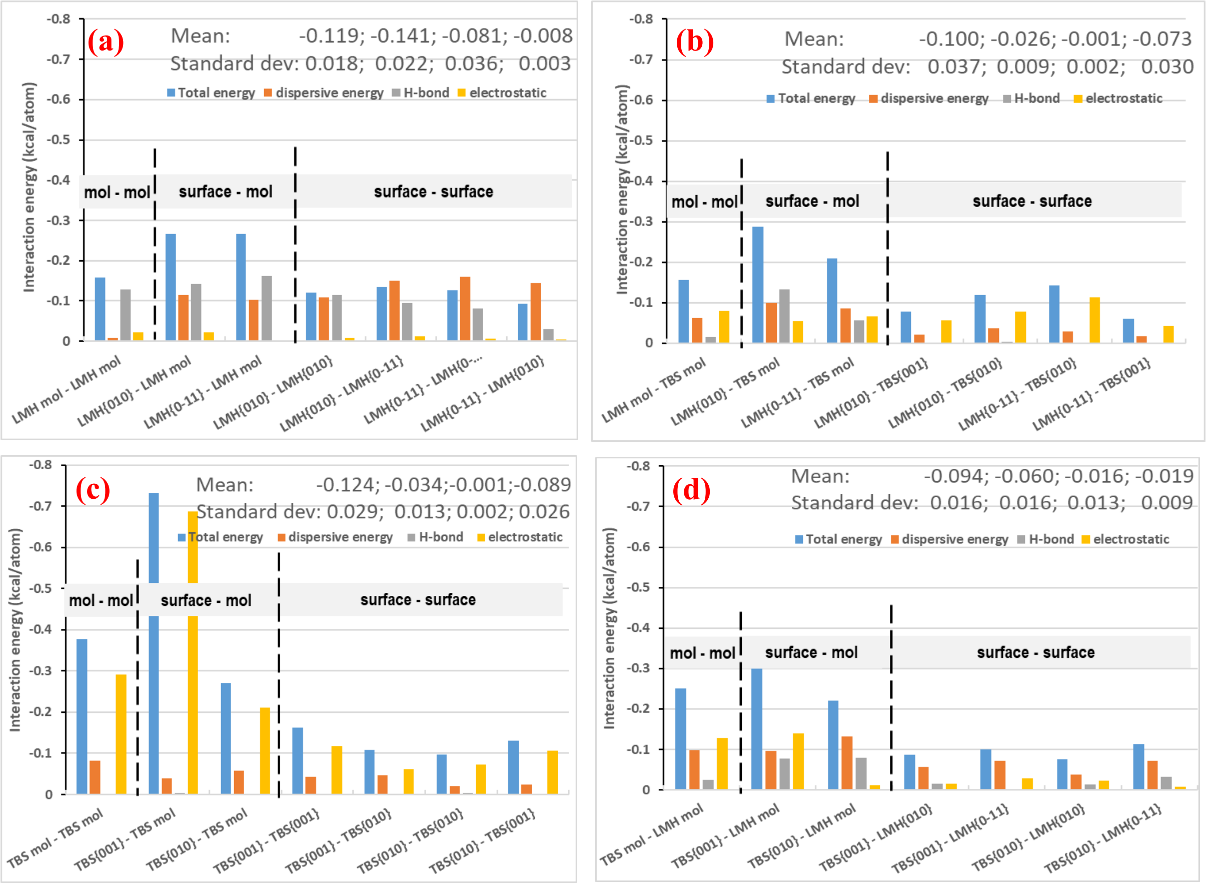

Figure S2. Total interaction, dispersive, hydrogen bonding and electrostatic energies of molecule and molecule, surface and molecule, and surface and surface of (a) LMH – LMH, (b) LMH – TBS; (c) TBS – TBS, and (d) TBS – LMH, with the mean energy and standard deviation for the four groups. Note that 1 kcal = 4.184 kJ.

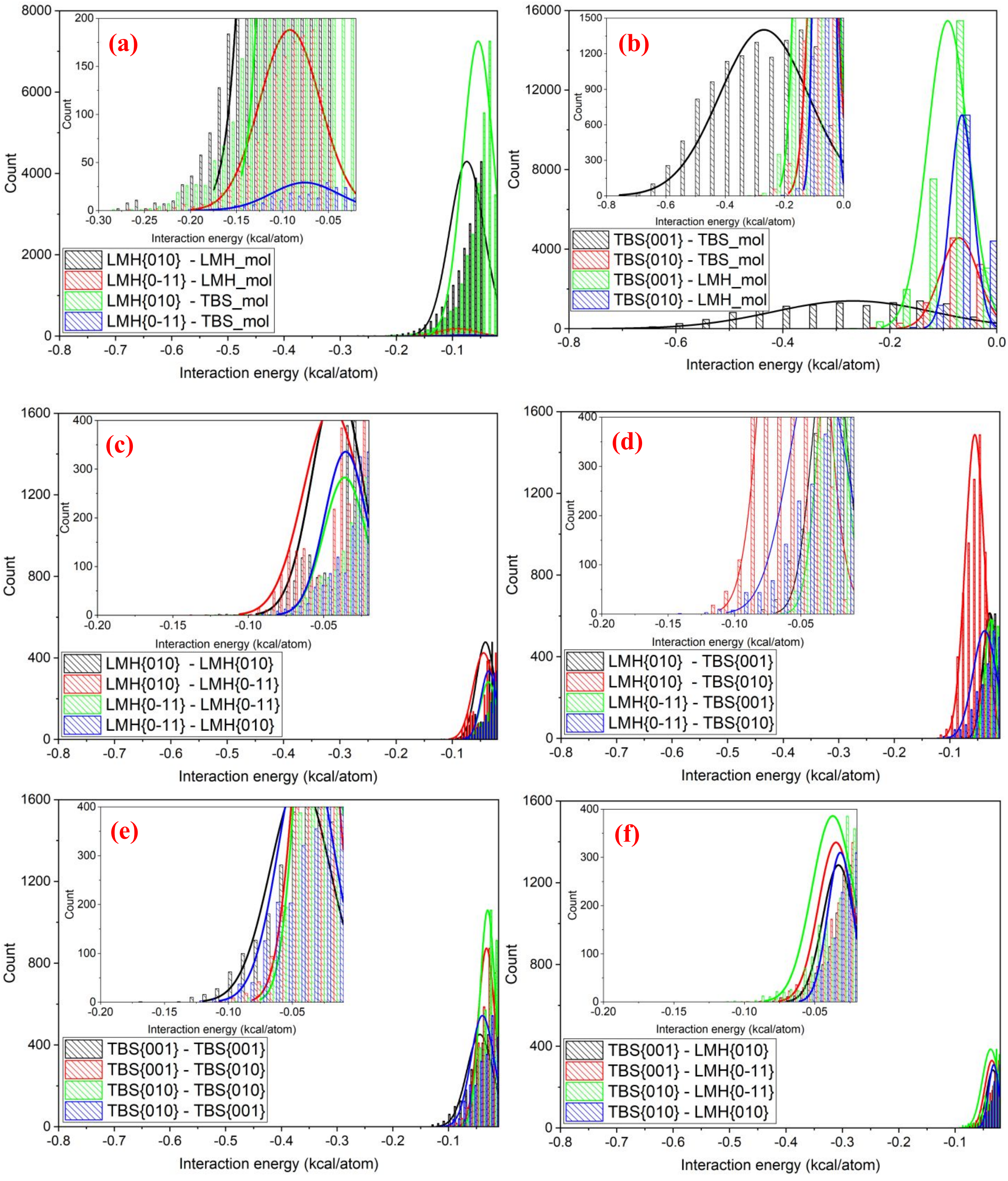

Figure S3. The interaction (binding) strength distributions of (a) LMH surfaces – LMH or TBS molecule, (b) TBS surfaces – TBS or LMH molecule, (c) LMH surface {010} – LMH or TBS surfaces, (d) LMH surface {0-11} – LMH or TBS surfaces, (e) TBS surface {001} – TBS or LMH surfaces, (f) TBS surface {010} – TBS or LMH surfaces, with the fitting curves using a Gaussian distribution function. Note that 1 kcal = 4.184 kJ.

Table S1. The systematic search results for interaction energy: TBS molecule – LMH molecule, TBS surface – LMH molecule and LMH surface – TBS molecule, TBS faces ({001}, {010}) - LMH faces ({010}, {0-11}).

SystSearch Methods	Host – Probe for SystSearch	Interaction Energy		Mean & Standard Deviation	
		kcal/atom	kJ/atom	kcal/atom	kJ/atom
**Molecule - Molecule**	TBS molecule – TBS molecule	-0.377	-1.577		
	LMH molecule – LMH molecule	-0.159	-0.665		
	TBS molecule – LMH molecule	-0.250	-1.046		
**Surface - Molecule**	TBS{001} – TBS molecule	-0.731	-3.059	-0.668 ± 0.309	-2.795 ± 1.293
	TBS{010} – TBS molecule	-0.270	-1.130		
	TBS{100} – TBS molecule	-0.382	-1.598		
	TBS{1-10} – TBS molecule	-0.332	-1.389		
	TBS{001} – LMH molecule	-0.313	-1.310	-0.311 ± 0.070	-1.301 ± 0.293
	TBS{010} – LMH molecule	-0.221	-0.925		
	TBS{100} – LMH molecule	-0.340	-1.423		
	TBS{1-10} – LMH molecule	-0.373	-1.561		
	LMH{010} – LMH molecule	-0.267	-1.117	-0.288 ± 0.026	-1.205 ± 0.109
	LMH{0-11} – LMH molecule	-0.267	-1.117		
	LMH{100} – LMH molecule	-0.335	-1.402		
	LMH{1-10} – LMH molecule	-0.271	-1.134		
	LMH{110} – LMH molecule	-0.298	-1.247		
	LMH{010} – TBS molecule	-0.287	-1.201	-0.267 ± 0.041	-1.117 ± 0.172
	LMH{0-11} – TBS molecule	-0.209	-0.874		
	LMH{100} – TBS molecule	-0.284	-1.188		
	LMH{1-10} – TBS molecule	-0.253	-1.059		
	LMH{110} – TBS molecule	-0.301	-1.259		
**Surface - Surface**	TBS{001} – TBS{001}	-0.162	-0.678	-0.124 ± 0.029	-0.519 ± 0.121
	TBS{010} – TBS{010}	-0.097	-0.406		
	TBS{001} – TBS{010}	-0.108	-0.452		
	TBS{010} – TBS{001}	-0.130	-0.544		
	TBS{001} – LMH{010}	-0.088	-0.368	-0.094 ± 0.016	-0.393 ± 0.067
	TBS{001} – LMH{0-11}	-0.100	-0.418		
	TBS{010} – LMH{010}	-0.075	-0.314		
	TBS{010} – LMH{0-11}	-0.113	-0.473		
	LMH{010} – LMH{010}	-0.120	-0.502	-0.119 ± 0.018	-0.498 ± 0.075
	LMH{0-11} – LMH{0-11}	-0.127	-0.531		
	LMH{010} – LMH{0-11}	-0.135	-0.565		
	LMH{0-11} – LMH{010}	-0.093	-0.389		
	LMH{010} – TBS{001}	-0.077	-0.322	-0.100 ± 0.037	-0.418 ± 0.155
	LMH{010} – TBS{010}	-0.119	-0.498		

	LMH{0-11} – TBS{001}	-0.061	-0.255		
	LMH{0-11} – TBS{010}	-0.142	-0.594		

Table S2. The means and standard deviations of interaction energy distributions after Gaussian fittings: LMH surfaces – LMH or TBS molecule, TBS surfaces – TBS or LMH molecule, LMH surface {010} – LMH or TBS surfaces, LMH surface {0-11} – LMH or TBS surfaces, TBS surface {001} – TBS or LMH surfaces, and TBS surface {010} – TBS or LMH surfaces.

**Search Methods**	**Host – Probe**	**Mean**		**Standard Deviation**	
		**(kcal/atom)**	**kJ/atom**	**(kcal/atom)**	**kJ/atom**
**Surface - Molecule**	LMH{010} – LMH molecule	-0.075	-0.314	0.031	0.130
	LMH{0-11} – LMH molecule	-0.092	-0.385	0.033	0.138
	LMH{010} – TBS molecule	-0.054	-0.226	0.028	0.117
	LMH{0-11} – TBS molecule	-0.076	-0.318	0.037	0.155
	TBS{001} – TBS molecule	-0.269	-1.126	0.150	0.628
	TBS{010} – TBS molecule	-0.070	-0.293	0.036	0.151
	TBS{001} – LMH molecule	-0.092	-0.385	0.038	0.159
	TBS{010} – LMH molecule	-0.066	-0.276	0.021	0.088
**Surface - Surface**	LMH{010} – LMH{010}	-0.041	-0.172	0.016	0.067
	LMH{010} – LMH{0-11}	-0.045	-0.188	0.019	0.079
	LMH{010} – TBS{001}	-0.028	-0.117	0.012	0.050
	LMH{010} – TBS{010}	-0.055	-0.230	0.017	0.071
	LMH{0-11} – LMH{0-11}	-0.035	-0.146	0.014	0.059
	LMH{0-11} – LMH{010}	-0.036	-0.151	0.014	0.059
	LMH{0-11} – TBS{001}	-0.027	-0.113	0.010	0.042
	LMH{0-11} – TBS{010}	-0.038	-0.159	0.021	0.088
	TBS{001} – TBS{001}	-0.044	-0.184	0.023	0.096
	TBS{001} – TBS{010}	-0.032	-0.134	0.015	0.063
	TBS{001} – LMH{010}	-0.033	-0.138	0.011	0.046
	TBS{001} – LMH{0-11}	-0.035	-0.146	0.012	0.050
	TBS{010} – TBS{010}	-0.030	-0.126	0.014	0.059
	TBS{010} – TBS{001}	-0.039	-0.163	0.021	0.088
	TBS{010} – LMH{010}	-0.032	-0.134	0.009	0.038
	TBS{010} – LMH{0-11}	-0.037	-0.155	0.015	0.063
